# Branching Out: Alterations in Bacterial Physiology and Virulence Due to Branched-Chain Amino Acid Deprivation

**DOI:** 10.1128/mBio.01188-18

**Published:** 2018-09-04

**Authors:** Julienne C. Kaiser, David E. Heinrichs

**Affiliations:** a Department of Microbiology and Immunology, University of Western Ontario, London, Ontario, Canada; University of Texas Health Science Center at Houston

**Keywords:** *Listeria*, *Staphylococcus*, branched-chain amino acids, nutrient starvation response, regulation, virulence

## Abstract

The branched-chain amino acids (BCAAs [Ile, Leu, and Val]) represent important nutrients in bacterial physiology, with roles that range from supporting protein synthesis to signaling and fine-tuning the adaptation to amino acid starvation. In some pathogenic bacteria, the adaptation to amino acid starvation includes induction of virulence gene expression: thus, BCAAs support not only proliferation during infection, but also the evasion of host defenses. A body of research has accumulated over the years to describe the multifaceted physiological roles of BCAAs and the mechanisms bacteria use to maintain their intracellular levels. More recent studies have focused on understanding how fluctuations in their intracellular levels impact global regulatory pathways that coordinate the adaptation to nutrient limitation, especially in pathogenic bacteria. In this minireview, we discuss how these studies have refined the individual roles of BCAAs, shed light on how BCAA auxotrophy might promote higher sensitivity to exogenous BCAA levels, and revealed pathogen-specific responses to BCAA deprivation. These advancements improve our understanding of how bacteria meet their nutritional requirements for growth while simultaneously remaining responsive to changes in environmental nutrient availability to promote their survival in a range of environments.

## Abstract (precis)

The branched-chain amino acids (BCAAs [Ile, Leu, and Val]) represent important nutrients in bacterial physiology, with roles that range from supporting protein synthesis to signaling and fine-tuning the adaptation to amino acid starvation. In some pathogenic bacteria, the adaptation to amino acid starvation includes induction of virulence gene expression: thus, BCAAs support not only proliferation during infection, but also the evasion of host defenses.

## BACTERIAL ADAPTATION TO NUTRIENT AVAILABILITY

Bacteria sense a variety of physical and chemical signals in their environment and use this information to coordinate an adaptive response that promotes their growth and survival. These signals include nutrients such as sugars, lipids, metals, and amino acids, and their depletion triggers upregulation of high-affinity nutrient acquisition systems and/or biosynthesis pathways. Nutrient availability is also an important environmental cue during infection, as pathogens encounter fluctuating nutrient access in a host and must adapt their metabolism accordingly to proliferate and avoid being cleared. Host environments impose an added challenge by actively sequestering nutrients to limit pathogen growth. To cope, pathogens release toxins that act to liberate nutrients from host tissues. In this way, virulence determinant production is tied to the nutrient composition of a given host niche.

Several transcriptional regulators are positioned at the intersection of metabolism and pathogenesis, such as CcpA (responds to a preferred carbon source), CodY (responds to GTP and branched-chain amino acids [BCAAs]), and RpiR (responds to pentose phosphate pathway intermediates) (1, 2). The relevance of such regulators to gross bacterial physiology is typically evidenced by mutating the regulator and measuring transcriptional changes to identify shifts in metabolism that presumably allow the pathogen to adapt to restriction of a given nutritional signal. This approach assumes that adaptive responses are binary, with each category defined by a completely active or inactive regulator. This methodology, however, fails to capture the intermediate responses that occur along a gradient of nutrient concentrations, which are more likely to represent the conditions pathogens encounter *in vivo*. It has remained challenging to capture the adaptive responses along a spectrum of nutrient availability because the mechanisms pathogens use to obtain nutrients, which therefore dictate this spectrum, are often unknown. Without a good understanding of the mechanisms that influence intracellular levels of regulatory nutrients, it remains largely unanswered how pathogens fine-tune intracellular nutrient pools at levels that are sufficient to promote growth in host tissues, while still allowing the bacteria to maintain sensitivity to changes in their external environment.

Several recent studies have applied a novel approach to address these gaps and have characterized the adaptive responses that correspond to graded nutritional levels (3–5). This approach, applied to the CodY regulator, involves mutating the effector binding site to mimic graded nutrient depletion, resulting in protein variants with fixed activation states along the full spectrum of CodY activity. Readers are directed to a recent review by Brinsmade (6) for a detailed summary of these studies. Comparison of the transcriptomes of cells bearing each protein variant has revealed that CodY-regulated genes are expressed as a hierarchy, whereby some target genes are derepressed upon a slight reduction of CodY activity, whereas other target genes remain repressed under the same conditions and are derepressed only upon a more significant reduction of CodY activity (3–5). These studies reveal the sensitivity of important physiological responses, including virulence gene expression, to changes in intracellular nutrient availability, namely BCAA concentrations. This has renewed interest in characterizing the factors that dictate intracellular BCAA concentrations, such as endogenous synthesis and acquisition mechanisms, and in investigating the relative contribution of these mechanisms to supplying BCAAs at key threshold concentrations that define a microorganism’s physiological state. In this review, we will discuss the roles of BCAAs in bacterial physiology, the mechanisms of biosynthesis and transport, and the recent advancements made in understanding how depletion of intracellular sources impacts pathogen proliferation and adaptation in host niches, with a focus on the role of BCAAs in regulating CodY. Although CodY is found only in Gram-positive bacteria, we will nonetheless discuss BCAA metabolism in both Gram-negative and Gram-positive bacteria to highlight shared physiological roles and mechanisms of acquisition and biosynthesis.

## MULTIFACETED ROLE FOR BRANCHED-CHAIN AMINO ACIDS IN BACTERIAL PHYSIOLOGY

The BCAAs are small nonpolar amino acids with branched alkyl side chains that make them hydrophobic and confer unique properties in proteins. Leu is a strong stabilizer of α-helical structures and, as such, is typically found in the inner helical core of proteins (7), whereas the substitution of the β-carbon with a methyl group on Ile and Val creates bulkiness that destabilizes α-helical structures; thus, Ile and Val are preferentially located in β-sheets (8, 9).

**FIG 1:**
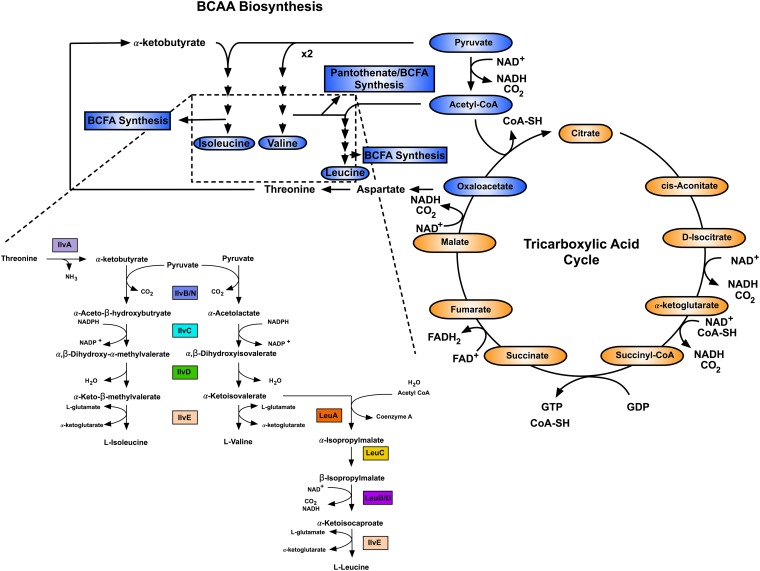
Integration of the BCAA biosynthetic pathway with cellular metabolism. Metabolites highlighted in blue are connected to BCAA biosynthesis.

Bacteria synthesize BCAAs through a conserved pathway that is present in fungi and plants, but absent in mammals. The level of synthesis is dependent on the availability of metabolites linked to central metabolism, including pyruvate, acetyl coenzyme A (acetyl-CoA), and oxaloacetate (Fig. 1). The biosynthetic pathway also provides intermediates for the synthesis of vitamin B_5_ (pantothenate) (10) and branched-chain fatty acids (BCFAs) (Fig. 1). BCFAs are the predominant fatty acids in Gram-positive bacterial membranes, and the nature and abundance of specific BCFAs determine the biophysical properties of the membrane (11). This contrasts with Gram-negative bacteria, where the predominant fatty acids are straight-chain fatty acids (SCFAs) and the biophysical properties of the membrane are determined by the degree of saturation (11). SCFA and BCFA synthesis proceeds through the same multienzyme fatty acid synthesis (FAS-II) pathway; however, the substrates that initiate the pathway differ. Acetyl-CoA serves as the substrate for SCFA synthesis, whereas branched-chain acyl-CoA serves as the substrate for BCFA synthesis (12). When initiated with branched-chain acyl-CoA substrates, the pathway produces even-chain anteiso-FAs derived from Ile, even-chain iso-FAs derived from Leu, and odd-chain iso-FAs derived from Val. Regulation of the ratio of iso to anteiso fatty acids and/or SCFA to BCFA facilitates adaptation to changes in temperature, pH, salinity, and CO_2_ (13–20). The positioning of the methyl group on the acyl chain of anteiso-FAs disrupts close packing of membrane lipids, promoting a more fluid membrane. This property is critical to adaptation to growth at low temperatures, during which the anteiso-FA (namely, a15:0) content is increased (13, 17, 18, 20–22). Thus, the importance of BCAAs for bacterial physiology stems from their integration with central metabolism, their requirement for protein synthesis, and their requirement for environmental adaptation via BCFA synthesis in Gram-positive bacteria.

## BCAAs AS INDICATORS OF CELLULAR METABOLIC STATUS

**FIG 2:**
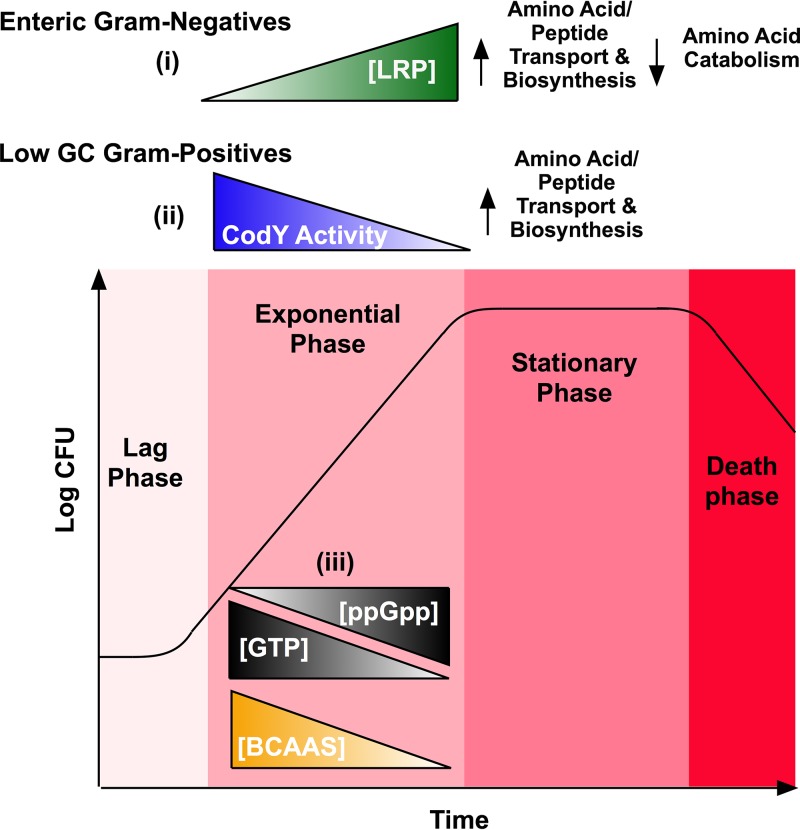
Involvement of BCAAs in the regulatory response to amino acid deprivation. Exponential growth is associated with nutrient consumption and subsequent depletion. Amino acid depletion triggers the synthesis of ppGpp from GTP, correlating with the entrance to stationary phase. Accumulation of ppGpp induces *lrp* expression in Gram-negative bacteria. (i) Leu binds to Lrp, genes involved in amino acid synthesis and transport are activated, and genes involved in amino acid catabolism are repressed. (ii) Depletion of GTP and BCAAs trigger a decrease in CodY DNA-binding activity in Gram-positive bacteria, and CodY target genes involved in amino acid biosynthesis and transport are expressed. (iii) BCAAs promote the hydrolysis activity of the enzyme that converts ppGpp to GTP, limiting either induction of *lrp* expression or inactivation of CodY.

In addition to their physiological roles, the BCAAs are effectors of the global transcriptional regulators leucine-responsive regulatory protein (Lrp) in Gram-negative bacteria and CodY in Gram-positive bacteria (23, 24). These global regulators coordinate the response to nutrient availability and regulate metabolic reprogramming to sustain growth upon nutrient exhaustion, as exemplified by the characteristic metabolic shift to stationary phase under laboratory growth conditions (Fig. 2). This transition coincides with accumulation of (p)ppGpp, a metabolite synthesized from GTP during the stringent response, a response provoked by amino acid starvation (25).

Lrp is a highly conserved transcriptional regulator in enteric bacteria and regulates gene expression upon entry into stationary phase (23). Lrp DNA-binding activity is enhanced, antagonized, or not affected by Leu availability, depending on the target gene, and binding can lead to either transcriptional repression or activation (26). Its target genes include those involved in glutamate, glutamine, BCAA, and serine biosynthesis, glycine degradation, BCAA and oligopeptide transport, and pilus formation (26). CodY, a conserved transcriptional regulator in low-GC Gram-positive bacteria (i.e., *Firmicutes*), senses the metabolic status of the cell to promote adaptation to nutrient limitation (27, 28). CodY is activated through direct interaction with BCAAs and GTP (27–30), with the exception of Lactococcus lactis and Streptococcus pneumoniae, where CodY responds to only BCAAs (31–33). In its active state, CodY binds to DNA, typically leading to transcriptional repression, although direct activation has been observed involving an unknown mechanism (34, 35). Thus, in general, CodY target genes are repressed during rapid growth and expressed upon nutrient deprivation. CodY regulates metabolic genes involved in amino acid synthesis, purine biosynthesis, sugar and amino acid transport, and the Krebs cycle (31, 36–39), as well as, in some species, sporulation (27, 40–42) and biofilm formation (43–45). CodY also regulates virulence gene expression in Gram-positive pathogens, including Bacillus anthracis (46–48), Bacillus cereus (43, 49–51), Clostridium difficile (40, 52, 53), Clostridium perfringens (41, 54, 55), Listeria monocytogenes (35, 39, 56, 57), Staphylococcus aureus (5, 37, 38, 58–60), S. pneumoniae (32), and Streptococcus pyogenes (45, 61–63). The role of CodY as a regulator of metabolism and virulence has been comprehensively reviewed elsewhere; thus, readers are directed to several recent reviews for more information (6, 24, 64, 65).

The accumulation of (p)ppGpp and consequential depletion of GTP impact the regulatory responses of Lrp and CodY, respectively, thereby linking these regulators to the stringent response (Fig. 2). Accumulation of (p)ppGpp induces *lrp* expression, and depletion of GTP decreases CodY DNA-binding activity (27–30, 66). In *Betaproteobacteria* and *Gammaproteobacteria*, the relative levels of (p)ppGpp and GTP are controlled by the synthetase RelA, which converts GTP to (p)ppGpp, and the hydrolase SpoT, which reverses the reaction (67). In other genera, a single bifunctional enzyme, RSH (RelA/SpoT homologue [or Rel]), interconverts (p)ppGpp and GTP (67). Recently, BCAAs were found to regulate the accumulation of (p)ppGpp (68). In *Alphaproteobacteria*, Val and Ile bind and activate the domain of the RSH that is responsible for hydrolyzing (p)ppGpp to GTP (68). Thus, the stringent response is countered under conditions of high BCAAs and promoted under conditions of BCAA limitation. This regulatory mechanism might also occur in other bacteria, as Val binds to the RelA enzyme in *Gammaproteobacteria* and Leu binds to the RSH enzyme in Gram-positive bacteria (68). Thus, BCAAs influence Lrp and CodY regulatory responses directly by binding and regulating their DNA-binding activity, and indirectly by regulating (p)ppGpp hydrolysis to GTP.

### An emerging role for isoleucine in regulating Gram-positive environmental adaptation.

Historically, all three BCAAs have been considered equal CodY effectors. Indeed, all three bind to CodY and activate its DNA-binding activity (29, 30, 69, 70), and yet, some studies have observed a stronger effect of Ile on CodY DNA-binding activity than either Leu or Val (31, 71–73). A predominant role for Ile in regulating CodY activity during growth has also emerged. Depletion of Ile and not Leu or Val relieves repression of CodY-regulated genes in B. subtilis (29), L. monocytogenes (57), and S. aureus (38, 69). Furthermore, as CodY target genes tend to comprise metabolic pathways, such as amino acid biosynthesis, the levels of Ile in the growth medium consequentially impact growth rate. Addition of excess Ile to the growth medium impairs growth of L. lactis (71), Streptococcus mutans (73), and S. aureus (38), whereas Ile depletion has the opposite effect. S. aureus growth is significantly impaired in media lacking Leu; however, its growth is restored upon simultaneous Ile and Leu depletion (69). An S. mutans mutant that is unable to synthesize (p)ppGpp (i.e., mimicking a strain with constitutively active CodY) is not able to grow in media lacking Leu or Val, but is able to grow in media lacking Ile, Leu, and Val (74). These growth restoration phenotypes are most likely CodY dependent, requiring Ile depletion to relieve CodY repression of amino acid biosynthesis. Given that the results of DNA-binding assays are influenced by the conditions tested (i.e., pH or salt [75]), the growth assays are likely a more accurate representation of intracellular conditions. As such, this would suggest that, inside the cell, Ile is the predominant BCAA to affect CodY activity. When considered in the context of Gram-positive pathogens, in which CodY also regulates virulence gene expression, this suggests that Ile could serve as a host metabolic cue. Indeed, Ile availability influences virulence gene expression in L. monocytogenes and S. aureus (discussed in more detail below) (38, 57, 69, 76). These advances have renewed interest in the factors that influence intracellular availability of Ile (and BCAAs in general) to better understand how pathogens scavenge BCAAs and where in the host they encounter BCAA limitation.

## FACTORS THAT INFLUENCE INTRACELLULAR BCAA AVAILABILITY

### BCAA biosynthesis.

BCAAs are synthesized through a conserved pathway in Gram-negative and Gram-positive bacteria (Fig. 1), with the exception of the BCAA auxotrophs Erysipelothrix rhusiopathiae, *Mycoplasma* spp., *Ureaplasma* spp., Peptostreptococcus anaerobius, Streptococcus pyogenes, and Streptococcus agalactiae (1). For detailed biochemical descriptions of each of the biosynthetic enzymes and regulation of their activity, readers are directed to a recent review by Amorim Franco and Blanchard (77).

The Leu biosynthetic genes are typically clustered in an operon (*leuABCD*) that may be either separate from the *ilv* genes or organized in a single *ilv*-*leu* operon, depending on the species. Several mechanisms of transcriptional regulation of these genes have been described, many involving transcriptional repression in response to BCAA availability. In Gram-negative bacteria, this is primarily mediated by attenuation. The attenuator region precedes the operon and is transcribed with the biosynthetic operon. It encodes a small BCAA-rich peptide, and as such, its rate of translation is determined by BCAA availability. When BCAA levels are high, the peptide is readily translated, allowing for a terminator secondary structure to form, which terminates transcription. When BCAA levels are low, the peptide is translated slowly, promoting an antiterminator secondary structure to form, allowing transcription to proceed. In Escherichia coli, the expression of many of the BCAA biosynthetic genes (organized as *ilvGMEDA*, *leuABCD*, *ilvC*, *ilvIH*, and *ilvBN*) is controlled by attenuation. The *ilvGMEDA* operon is preceded by a 32-amino-acid (aa) peptide that contains 15 BCAAs (78, 79), *ilvBN* is preceded by a 32-aa peptide with 11 BCAAs (80), and the *leuABCD* operon is preceded by a 28-aa peptide containing 4 Leu (81). Attenuation is also the primary mechanism of regulating the *leu* operon in Salmonella enterica serovar Typhimurium (82, 83) and putative leader peptides and terminator hairpins are found upstream of BCAA biosynthesis genes across various Gram-negative species (84), suggesting that BCAA-dependent attenuation is a conserved mechanism. The biosynthetic genes in E. coli are also regulated by Lrp, which binds to the *ilvIH* and *ilvG* promoters in the absence of Leu to activate and repress transcription, respectively (85–87).

In Gram-positive bacteria, multiple global regulators coordinate expression of the BCAA biosynthetic operon in response to not only BCAA availability but also carbon and nitrogen availability (88). In B. subtilis, the single *ilv-leu* operon is positively regulated by the carbon catabolite protein A (CcpA) (88, 89), a global transcriptional regulator that regulates carbon utilization in response to a preferred carbon source (90). This positive regulation is antagonized by either TnrA, a regulator that responds to nitrogen limitation, or CodY (91). Together, this allows for conservation of carbon and nitrogen when exogenous BCAA sources are present. Additional fine-turning of *ilv-leu* expression is mediated by a Leu-responsive T-box riboswitch (88, 92–94), as well as mRNA processing (95). CodY-dependent repression of BCAA biosynthesis is common across Gram-positive bacteria (32, 37, 38, 51, 52, 56), whereas TnrA homologues are less conserved (96). In contrast to the tRNA-mediated attenuation observed in B. subtilis, recent experimental evidence implicates ribosome-mediated attenuation as an important mechanism regulating BCAA biosynthesis in S. aureus and L. monocytogenes (69, 76). Furthermore, bioinformatics analysis of the leader sequence of the BCAA biosynthetic genes in L. lactis (97), Corynebacterium glutamicum (98, 99), and *Streptococcus* spp. (100) have revealed BCAA-rich peptides and terminator hairpins consistent with attenuation, suggesting that ribosome-mediated attenuation is a common mechanism controlling transcription of the BCAA biosynthetic operon in Gram-positive bacteria. In S. aureus, an additional regulatory mechanism governing BCAA biosynthesis has been described involving repression by the essential Gcp/YeaZ complex (101, 102). YeaZ binds upstream of the *ilv-leu* operon, suggesting possible direct repression of the operon (102), although the conditions that regulate its binding are currently unknown.

### The BCAA auxotrophy paradox.

Even in the presence of intact biosynthesis genes, some Gram-positive bacteria synthesize little to no BCAAs in the absence of an exogenous source, with some even being misclassified as auxotrophs. L. monocytogenes synthesizes very little Ile, Leu, and Val despite possessing intact and functional biosynthetic genes (103, 104). Similarly, S. pneumoniae is unable to grow in a chemically defined medium when Ile, Leu, or Val is omitted (105). Streptococcus suis exhibits a Leu auxotrophy despite possessing intact Leu biosynthesis genes and synthesizes moderate levels of Val but no detectable Ile (106). Despite possessing an intact BCAA biosynthetic operon, S. aureus exhibits a significant growth delay in the absence of Leu, and growth in the absence of Val occurs only upon accumulation of suppressor mutations (69, 107). One possible explanation for an observed “auxotrophy” could be the availability of biosynthetic precursors. L. monocytogenes lacks a 2-oxoglutarate dehydrogenase enzyme and is therefore unable to derive oxaloacetate from the tricarboxylic acid (TCA) cycle (Fig. 1). Instead, L. monocytogenes carboxylates pyruvate to form oxaloacetate in environments where glucose is the sole carbon source (103). Shortage of this indirect BCAA precursor might explain the limited amounts of BCAAs synthesized in this species (103, 104). Another possible factor could be tight transcriptional repression of the biosynthetic operon. S. aureus is able to grow in the absence of Val only upon selection of strains with mutations that relieve either CodY-dependent repression or attenuator-dependent repression (69), indicating that the *ilv-leu* operon remains under tight repressive control even in the absence of Val. Repression is relieved upon Ile depletion via CodY and to a lesser extent, upon Leu depletion via transcriptional attenuation (69). Such tight control is also observed in L. monocytogenes, which also regulates BCAA biosynthesis in response to Ile via CodY-dependent repression and a BCAA-rich attenuator peptide (76).

### Specialized BCAA transporters.

The repression of the BCAA biosynthetic genes in response to BCAA availability allows for conservation of carbon and nitrogen when an exogenous source can be acquired. Active transporters specific to BCAAs are common across bacteria and require either ATP or the proton motive force to import BCAAs. BCAA transporters in Gram-negative bacteria include the high-affinity LIV-I system and the low-affinity LIV-II and LIV-III systems (Table 1). LIV-I is an ATP-binding cassette (ABC) transporter encoded by *livJKHMGF* (108). Two substrate binding proteins mediate BCAA transport through this system; LIV-B (*livJ*), which can bind all three BCAAs, and LS-B (*livK*), which is Leu specific (109, 110). LIV-II, also known as BrnQ, is a permease with 12 transmembrane helices and belongs to the major facilitator superfamily (MFS). LIV-III is a permease homologous to LIV-II, with transport activity that is obviated only in a LIV-II deficient background in *Salmonella* Typhimurium and Pseudomonas aeruginosa (111, 112). LIV-II and LIV-III use energy from the proton motive force to couple BCAA transport with Na^+^ across an energy gradient (113). An additional MFS transporter specific to Ile transport and unique to Francisella tularensis has 12 transmembrane helices and belongs to the phagosomal nutrient transporter family of MFS transporters identified in Legionella pneumophila (114, 115).

**TABLE 1:** BCAA transporters

Organism	Transporter	Energy source	Specificity^*a*^	Reference(s)
Gram-negative bacteria				
Chlamydia trachomatis	LIV-II (*brnQ*)	PMF^*b*^	ILV	156
Escherichia coli	LIV-I (*livKHMGF*)	ATP	L	108–110, 157
	LIV-I (*livJHMGF*)	ATP	ILV	
	LIV-II (*brnQ*)	PMF	ILV	
Francisella tularensis	*ileP*	PMF	I	114
Pseudomonas aeruginosa	LIV-I	ATP	ILV	158–162
	LIV-II (*braB*)	PMF	ILV	
	LIV-III (*braZ*)	PMF	ILV	
*Salmonella* Typhimurium	LIV-I	ATP	ILV	111, 163–166
	LIV-II (*brnQ*)	PMF	ILV	
	LIV-II	PMF	ILV	

Gram-positive bacteria				
Bacillus subtilis	*bcaP*	PMF	ILV	118
	*braB*	PMF	ILV	
	*brnQ*	PMF	ILV	
Corynebacterium glutamicum	*brnQ*	PMF	ILV	122
Lactobacillus delbrueckii	*brnQ*	PMF	ILV	121
Lactococcus lactis	*brnQ*	PMF	ILV	116, 117
	*bcaP*	PMF	ILV	
Staphylococcus aureus	*brnQ1*	PMF	ILV	107, 119
	*brnQ2*	PMF	I	
	*brnQ3*	NA^*c*^	NA	
	*bcaP*	PMF	ILV	
Streptococcus pneumoniae	*livAJHMGF*	ATP	ILV	120

a I, isoleucine; L, leucine; V, valine.

b PMF, proton motive force.

c NA, not applicable.

BrnQ also functions as a BCAA transporter in Gram-positive bacteria, along with a second nonhomologous permease, BcaP (Table 1). L. lactis acquires BCAAs via both BcaP and BrnQ, with BcaP playing a more predominant role (116, 117). Similarly, BcaP is the predominant transporter in B. subtilis, with two additional transporters, BrnQ and BraB (a BrnQ homologue), contributing to Ile and Val uptake and an unidentified transporter contributing to Leu uptake (118). In contrast, BrnQ1 serves as the predominant transporter for S. aureus growth, with BcaP playing a secondary role (107). S. aureus encodes two additional *brnQ* homologues: BrnQ2, an Ile-dedicated transporter, and BrnQ3, which has no observed BCAA transport function (107, 119). A LIV-I system with a substrate binding protein able to bind BCAAs has been described in S. pneumoniae, although no transport function has yet been ascribed to this system (120). BrnQ also directs BCAA transport in Lactobacillus delbrueckii and C. glutamicum (121, 122).

## BCAAS AT THE CROSSROADS OF METABOLISM AND VIRULENCE

### Mechanisms that support growth during infection.

BCAA availability at various infection sites remains undefined; however, both BCAA biosynthesis and transport have been linked to promoting the virulence of pathogens during infection, suggesting that pathogens encounter BCAA limitation *in vivo* (120, 123–129). Concentrations of BCAAs have been estimated in some host environments relevant to pathogens. Levels of BCAAs in the bloodstream range from 20 to 92 µM for Ile, 40 to 250 µM for Leu, and 65 to 266 µM for Val (130). Human nasal secretions contain Leu levels in the range of 130 to 287 µM, Val levels in the range of 13 to 156 µM, and very little or no Ile (131). Indeed, some pathogens exploit these extracellular BCAA sources during infection. S. aureus requires both the BrnQ1 and BcaP transporters for optimal fitness during systemic infection and nasal colonization (107, 119). BCAA acquisition also likely contributes to S. aureus lung infection, as transport genes are upregulated in a pneumonia model (132). The contribution of BCAA biosynthesis to S. aureus growth *in vivo* remains to be determined, although biosynthesis likely plays a role in maintaining BCAA levels, as the biosynthetic genes are upregulated when S. aureus is grown in blood, the lung environment, and in nasal secretions (131–133). In S. pneumoniae, BCAA transport supports growth in a systemic infection model, pneumonia model, and meningitis model, but not in a colonization model (120, 129), whereas BCAA biosynthesis is required for invasion of host tissue following intranasal colonization, but is not required for systemic infection (134). BCAA transport also contributes to growth of Yersinia pestis during systemic infection (128). Despite some pathogens being able to exploit extracellular BCAAs, some pathogens require BCAA biosynthesis for infection, including Klebsiella pneumoniae, Neisseria meningitidis, and P. aeruginosa (123, 124, 135).

Intracellular pathogens face the challenge of direct competition with the host for intracellular BCAAs since they are essential nutrients in humans. Indeed, several intracellular pathogens, including Burkholderia pseudomallei, Mycobacterium bovis, Mycobacterium tuberculosis, and L. monocytogenes rely on BCAA biosynthesis for replication inside host cells (136–140). Yet, some intracellular pathogens are auxotrophic for BCAAs and therefore necessitate transporters to obtain BCAAs. The BCAA auxotroph Legionella pneumophila requires the Val transporter PhtJ for intracellular growth (115, 141), and pathogenic subspecies of *Francisella*, which have lost the capacity to synthesize BCAAs, require the Ile transporter IleP for intracellular replication and infection *in vivo* (114). Interestingly, F. tularensis has been observed to induce a transient increase in cytosolic BCAA concentrations following infection, suggesting that it might manipulate host metabolism to support its own growth during infection (114).

If deprived of BCAAs, Gram-positive pathogens face challenges not only in supporting protein synthesis and growth, but also in maintaining the appropriate BCFA content to protect against host defenses that target the bacterial membrane. A role for BCFA synthesis in promoting resistance to host defenses is best highlighted in L. monocytogenes, where BCFAs comprise 75 to 98% of the membrane (13, 14, 16, 21, 142). BCFA-deficient strains have increased susceptibility to antimicrobial peptide killing and lysozyme digestion and decreased production of the virulence factor listeriolysin O (143), all of which likely contribute to the decreased intracellular growth and virulence of a strain deficient of BCFAs (143, 144). In S. aureus, BCFAs comprise 44 to 63% of the membrane and a BCFA-deficient strain exhibits reduced adherence to host cells and is attenuated *in vivo* (107, 145, 146).

While these studies reveal that BCAA deprivation limits pathogen metabolism and physiology *in vivo*, more research is needed to elucidate the relative importance of biosynthesis versus acquisition in various host niches and how this source preference might be regulated. One mechanism governing source preference involves positioning of the gene in the hierarchy of the CodY regulon. The BCAA biosynthetic operon and the transporter genes in B. subtilis are both controlled by CodY. CodY binds to a 15-nucleotide (nt) binding motif, AATTTTCWGAAAATT (71, 147), and nucleotide substitutions in the motif that deviate from the consensus sequence decrease the binding affinity of CodY (147, 148). The binding strength of the motif thus correlates with the extent of repression and dictates the positioning of the gene within the hierarchy of graded target gene derepression (3). In B. subtilis, BCAA biosynthesis and transport are derepressed at similar points along the spectrum of CodY activity (3), but in S. aureus, in which this hierarchical response is also observed, the BCAA transporter *brnQ2* is derepressed upon modest decreases in CodY activity, whereas BCAA biosynthesis remains repressed at this same level of CodY inactivation (5), suggesting that S. aureus prioritizes nutrient scavenging upon modest nutrient depletion to prevent unnecessary divergence of carbon and nitrogen to nutrient synthesis. The positioning of other transcriptional regulators in the CodY hierarchy adds an additional layer of metabolic fine-tuning. For example, CodY is a direct repressor of *braB*, a BCAA transporter in B. subtilis, but it is a stronger repressor of *scoC*, which also represses *braB* expression. *braB* expression is therefore optimal at intermediate levels of CodY activity, when CodY-dependent *braB* repression is partially relieved, and *scoC* remains repressed (149). Such precise sensitivity to CodY activity represents one way by which pathogens can fine-tune the coordination of nutrient acquisition strategies.

### BCAAs as host cues to regulate virulence.

**FIG 3:**
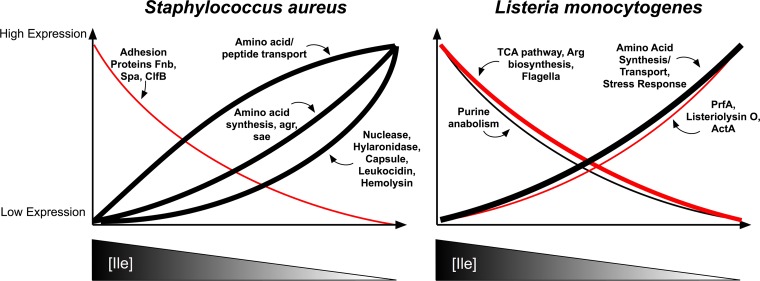
CodY regulation of virulence genes in Staphylococcus aureus and Listeria monocytogenes. CodY functions primarily as a repressor in S. aureus, and its target genes are repressed in the presence of Ile and expressed as a hierarchy upon Ile depletion (black line). Some target genes are activated by CodY in S. aureus and are expressed in the presence of Ile and repressed upon Ile depletion (red line). In L. monocytogenes, CodY functions as both an activator and a repressor under both high- and low-Ile conditions. Under high-Ile conditions, CodY acts as an activator (red line). CodY also functions as an activator under low-Ile conditions (red line) and induces expression of virulence genes. Black lines indicate genes that are repressed by CodY, and red lines indicate genes that are activated by CodY. The thickness of the line corresponds to the relative proportion of genes in that category.

The predominant role of CodY as a negative regulator of virulence and the hierarchical regulation of CodY target genes in S. aureus is but one example of how pathogens respond to BCAA starvation and adapt to their environment. In some pathogens, it has been shown that CodY can also function as a positive regulator. Table 2 outlines the positive and/or negative regulatory role of CodY on notable virulence factors in several Gram-positive pathogens. To fully appreciate the complexity of pathogen-specific CodY responses, this section will contrast the predominant repressive role of CodY in S. aureus to the complex role of CodY as both a positive and negative regulator of virulence in L. monocytogenes (summarized in Fig. 3). The advancements made in these pathogens reveal that pathogen lifestyle might have influenced the regulatory response coordinated in response to BCAA availability.

**TABLE 2:** Regulation of virulence by CodY in Gram-positive pathogens

Organism	Phenotype of *codY* mutant *in vivo*	Notable virulence gene regulation	Reference(s)
Staphylococcus aureus	Hypervirulent in murine skin abscess and pneumonia; no effect on systemic infection	Indirect repression of delta-toxin/RNAIII via repression of *agr* activator; direct repression of biofilm synthesis (*icaADBC*), alpha-toxin (*hla*), hyaluronidase (*hysA*), Panton-Valentine leucocidin (*lukSF-PV*)	37, 59, 167, 168
Streptococcus pneumoniae	Reduced colonization; no effect on systemic infection	Direct activation of adhesion protein choline-binding protein (*pcpA*)	32
Bacillus anthracis	Attenuated virulence in murine toxinogenic model	Indirect activation of anthrax toxin components (*cya*, *lef*, *pagA*) and direct repression of S layer proteins (*sap*, *eag*) via AtxA; activation of iron scavenging systems	46–48
Clostridium perfringens			
Type D	NT^*a*^	Direct and indirect activation of epsilon toxin (ETX); repression of sporulation	41, 54
Type A		Activation of sporulation and enterotoxin (CPE)	55
Bacillus cereus (F4810/72)	Attenuated virulence in Galleria mellonella infection model	Indirect activation of cytotoxin (*cytK*), enterotoxin (*nhe*), and hemolysin (*hbl*) via direct activation of regulator *plcR*; direct repression of cereulide (*cesPTABCD*) and inhibitor metalloprotease 1 (*inhA1*)	50
Clostridium difficile	NT	Indirect repression of toxin A (*tcdA*) and B (*tcdB*) via direct repression of *tcdR*	53
Listeria monocytogenes	Attenuated virulence in murine systemic infection model	Indirect activation of listeriolysin O (*hyl*) via direct activation of regulator *prfA*; direct activation of flagellar biosynthesis and ActA	35, 39, 56, 57
Streptococcus pyogenes	NT	Indirect activation of surface proteins via activation of regulator *mga*; activation of regulators *fasX* and *pel*/*sagA*	61, 62

a NT, not tested.

In S. aureus, CodY binds to DNA under BCAA-replete conditions and primarily acts as a repressor of virulence genes (5, 37, 38). CodY regulates approximately 5% of the S. aureus genome, with the majority of its targets (85%) subject to repression by CodY (37, 38). These targets include virulence genes, such as the capsule genes, α-hemolysin and adhesion protein genes, as well as regulators of virulence gene expression, including the *agr* locus and *saeRS* two-component system (60, 150). Most of the virulence genes are directly repressed by CodY, whereas others, including the capsule genes and hemolysin genes, are indirectly activated through *agr* (37, 38). The genes activated by CodY, which fall into the categories of nucleotide transport/metabolism and adhesion proteins, do not have a CodY binding sequence, suggesting an indirect mechanism of regulation (37). The coordination of virulence gene expression with the environment is crucial for S. aureus to limit unwanted host damage, as exemplified by the hypervirulence of a *codY* mutant in a skin abscess and pneumonia model of infection (59). To ensure the appropriate expression of virulence genes, the CodY regulon is expressed as a hierarchy that depends on the extent of CodY activation and therefore nutrient (e.g., Ile) availability (Fig. 3) (5). The graded response prioritizes expression of amino acid and peptide transport over synthesis upon modest nutrient limitation and reserves expression of hydrolytic enzyme and toxin production for more severe nutrient limitation (Fig. 3) (5). Also within this spectrum are other virulence gene regulators, including the *agr* and *sae* loci, which together form a regulatory cascade that integrates several environmental cues, such as growth phase and host defenses (5, 150, 151). Together, the graded response and regulatory cascade are thought to maximize nutrient acquisition while limiting host toxicity (5).

In contrast to S. aureus, CodY in L. monocytogenes can bind to DNA under both BCAA-replete and BCAA-depleted conditions and can function as both a repressor and activator (35, 39). CodY directly or indirectly regulates approximately 14% of L. monocytogenes genes. Approximately 66% of these genes are upregulated in a *codY* mutant compared to the wild-type strain when grown in nutrient-rich medium (i.e., BCAA replete), consistent with the role of CodY as a repressor (Fig. 3) (39). The repressed genes are primarily involved in nutrient metabolism and transport and stress responses and include some virulence factors. The remaining 33% of differentially regulated genes that are downregulated in a *codY* mutant revealed an underappreciated role for CodY as an activator in this organism (39). The genes that are activated by CodY under these conditions include the arginine biosynthesis pathway and flagellar biosynthesis genes. Interestingly, CodY also acts as an activator under BCAA-depleted conditions, with approximately 30% of differentially regulated genes downregulated in a *codY* mutant compared to the wild-type strain when grown in BCAA-limited growth medium (Fig. 3). Under BCAA-depleted conditions, CodY is a direct activator of *prfA*, a global virulence gene regulator in L. monocytogenes (35). This leads to activation of PrfA-regulated virulence factors, including listeriolysin O and the surface protein ActA, which are important for intracellular replication and cell-to-cell spread, respectively (57, 152, 153). Consequently, an L. monocytogenes *codY* mutant is impaired in motility and intracellular replication (35, 39). The role of CodY during L. monocytogenes infection is more difficult to discern, because although a *codY* mutant is attenuated *in vivo* in comparison to a wild-type (WT) strain, implicating CodY as an activator of virulence, a *codY* mutation rescues the virulence of a strain where CodY is constitutively active (i.e., a *relA* mutant), also demonstrating its role as a repressor of virulence (35, 56). It is therefore challenging to classify CodY as either a repressor or activator of virulence in this organism. Rather its overall impact on virulence will depend on the resulting gene expression profile at any given CodY activation state.

The advancements made in these pathogens highlight how BCAA (namely, Ile) limitation is linked to promoting virulence of both organisms but via distinct mechanisms: that is, via CodY-dependent repression of virulence genes in S. aureus and CodY-dependent activation and/or repression of virulence genes in L. monocytogenes (5, 39). As such, virulence is significantly influenced by CodY activity, such that two distinct lifestyles (i.e., nontoxic versus toxic for S. aureus and motile versus cytosolic replication for L. monocytogenes) are displayed at either end of the spectrum of BCAA concentrations, suggesting that even modest changes in intracellular Ile concentrations can have drastic consequences for virulence. It is not surprising, then, that two recent studies have uncovered that both pathogens tightly regulate BCAA biosynthesis via a shared mechanism resulting in a BCAA “auxotrophy” phenotype, which might allow them to increase their capacity to respond to a wider range of BCAA levels to reduce the likelihood of untimely virulence determinant expression (69, 76).

### BCAA auxotrophy: a metabolic strategy to promote environmental adaptation?

As discussed in a previous section, both S. aureus and L. monocytogenes require the addition of BCAAs to the growth medium to support growth due to minimal levels of BCAA biosynthesis (69, 104, 107, 119). Two mechanisms of repression control this: (i) Ile-dependent CodY repression and (ii) a *cis-*acting BCAA-dependent attenuator (69, 76). CodY represses the attenuator and the *ilv-leu* operon under high-Ile conditions to limit BCAA biosynthesis. As Ile is depleted, repression is relieved; however, the attenuator further regulates the levels of *ilv-leu* transcripts in response to Leu and Ile availability in S. aureus and all three BCAAs in L. monocytogenes (69, 76). This additional “checkpoint” in repression is thought to delay the repletion of BCAAs, extending the range of BCAA starvation and therefore the range of CodY activation states. Indeed, an L. monocytogenes strain lacking the attenuator-dependent repression synthesizes more BCAAs and exhibits reduced expression of the virulence gene regulator *prfA* in comparison to the wild-type strain (76); that is to say, prompt repletion of BCAAs via endogenous synthesis prevents the cells from reaching a state of BCAA deprivation necessary for CodY to bind to the *prfA* promoter and activate its transcription. As discussed previously, several Gram-positive bacteria display little to no BCAA biosynthesis, suggesting that this might represent a metabolic strategy to better coordinate virulence gene expression in response to nutritional cues in the environment.

## CONCLUSIONS AND FUTURE DIRECTIONS

Recent advancements in the area of BCAA metabolism have identified the importance of BCAA acquisition and synthesis for pathogen growth *in vivo* and have revealed that a pathogen’s preferred strategy reflects its unique physiological needs and host tissue preferences. An emerging theme is that regulation of nutrient source preference is critical to maintaining tight control over intracellular concentrations of BCAAs and ensures that pathogens are responsive to fluctuations in these levels and therefore are able to initiate the appropriate adaptive response, which can have significant consequences for virulence. Therefore, future studies should continue to focus on identifying how each of these mechanisms influences intracellular pools of BCAAs. In Gram-positive bacteria, this includes evaluating how manipulation of BCAA transporters and/or biosynthesis influences intracellular levels of Ile and, subsequently, CodY activity. In S. aureus, depletion of exogenous Ile has a significant impact on CodY activity, and therefore CodY target genes are derepressed in a strain lacking the Ile transporter BrnQ2 (69). Similarly, mutation of BCAA transporters in B. subtilis leads to a decrease in CodY activation, although the sole contributions of each of the three transporters remain to be determined (118). Levels of endogenous synthesis, too, impact CodY activity in L. monocytogenes and B. subtilis (29, 76). In Gram-negative bacteria, the role of BCAA deprivation in regulating virulence remains to be explored. Evidence in the Gram-negative swine pathogen Actinobacillus pleuropneumoniae suggests the existence of a parallel response. A. pleuropneumoniae encounters BCAA limitation in the porcine lung and requires BCAA biosynthesis for full virulence in this environment (154). Furthermore, BCAA deprivation triggers upregulation of not only BCAA biosynthesis, but also several genes that are upregulated during infection, some of which have putative Lrp binding sites (155). This suggests that BCAA deprivation, sensed via Lrp, might also act as an important environmental cue for Gram-negative pathogens. Future studies will provide more insight into how pathogens obtain BCAAs and how they regulate their intracellular levels to promote their survival and ability to cause disease.
